# Moving Beyond the Brain: Transcutaneous Spinal Direct Current Stimulation in Post-Stroke Aphasia

**DOI:** 10.3389/fneur.2017.00400

**Published:** 2017-08-08

**Authors:** Paola Marangolo, Valentina Fiori, Jacob Shofany, Tommaso Gili, Carlo Caltagirone, Gabriella Cucuzza, Alberto Priori

**Affiliations:** ^1^Dipartimento di Studi Umanistici, Università degli Studi di Napoli Federico II, Napoli, Italy; ^2^IRCCS Fondazione Santa Lucia, Roma, Italy; ^3^Centro Fermi - Museo storico della fisica e Centro studi e ricerche Enrico Fermi, Rome, Italy; ^4^Università degli Studi di Roma Tor Vergata, Roma, Italy; ^5^Clinica Neurologica III, Dipartimento di Scienze della Salute, Università degli Studi di Milano, Milan, Italy

**Keywords:** transcutaneous spinal direct current stimulation, spinal cord, neurostimulation, aphasia, stroke, verb recovery

## Abstract

Over the last 20 years, major advances in cognitive neuroscience have clearly shown that the language function is not restricted into the classical language areas but it involves brain regions, which had never previously considered. Indeed, recent lines of evidence have suggested that the processing of words associated to motor schemata, such as action verbs, modulates the activity of the sensorimotor cortex, which, in turn, facilitates its retrieval. To date, no studies have investigated whether the spinal cord, which is functionally connected to the sensorimotor system, might also work as an auxiliary support for language processing. We explored the combined effect of transcutaneous spinal direct current stimulation (tsDCS) and language treatment in a randomized double-blind design for the recovery of verbs and nouns in 14 chronic aphasics. During each treatment, each subject received tsDCS (20 min, 2 mA) over the thoracic vertebrae (10th vertebra) in three different conditions: (1) anodic, (2) cathodic and (3) sham, while performing a verb and noun naming tasks. Each experimental condition was run in five consecutive daily sessions over 3 weeks. Overall, a significant greater improvement in verb naming was found during the anodic condition with respect to the other two conditions, which persisted at 1 week after the end of the treatment. No significant differences were present for noun naming among the three conditions. The hypothesis is advanced that anodic tsDCS might have influenced activity along the ascending somatosensory pathways, ultimately eliciting neurophysiological changes into the sensorimotor areas which, in turn, supported the retrieval of verbs. These results further support the evidence that action words, due to their sensorimotor semantic properties, are partly represented into the sensorimotor cortex. Moreover, they also document, for the first time, that tsDCS enhances verb recovery in chronic aphasia and it may represent a promising new tool for language treatment.

## Introduction

Since the late nineteenth century, it has long been assumed that the language function is hierarchically organized into specific cortical areas, the Broca’s and Wernicke’s areas (1). However, over the past decades, several lines of evidence have shown that the language faculty not only engages a number of cortical and subcortical regions that extend far beyond the classical areas [for reviews, see for example (2, 3)] but it is also represented within regions that had never been considered before to support language [for review see Ref. (4)]. Accordingly, instead of considering the language faculty as completely modularized, behavioral and neuroimaging results have shown that the network subserving the language function is largely distributed across the brain (5, 6). In the embodied cognition view, the representation of a concept is crucially dependent upon the sensory–motor properties belonging to that concept (7–9). Indeed, the hypothesis has been advanced that action verbs are mentally represented in different semantic representations among which the sensorimotor features to perform the action (10–12). This implies that the sensory–motor regions of the brain may also process action concepts. Several lines of evidence have already suggested that the sensorimotor cortex takes part in language processing, at least when speech is translated into sensorimotor acts (10, 13, 14). Indeed, much of this evidence comes from studies that used action verbs (individually presented or embedded in sentences) as stimuli [e.g., Ref. (13, 15, 16)]. Accordingly, when people listen to verbal description of actions, their somatosensory, motor, and premotor neural populations are activated as they are actually performing the corresponding actions [5–3, for reviews see Ref. (4, 17)]. Recently, slower hand motor responses have been shown during processing of nouns referring to hand-related objects [(11, 12); see also Ref. (18)].

Thus, the retrieval of words associated with motor schemata, like *swimming*, is thought to rely in part in sensorimotor regions of the brain. Similarly, manipulable nouns (i.e., a pen), which recruit motor representations (i.e., writing), are partly processed in the same regions (11, 12).

Together with this more interactive view of language processing, the traditional concept of the spinal cord as a hardwired system that automatically respond to motor commands from the brain and to sensory inputs from the periphery has changed over time. Indeed, a large body of evidence has shown that this structure not only produces a variety of specialized movements but also acquires and memorizes new behaviors [see Ref. (19) for review].

However, in spite of a remarkable energy in considering the spinal cord as an active system which possesses capacities for neuronal and synaptic plasticity for the recovery of chronic pain and motor deficits (20, 21), as far as we know, no studies have considered whether it might also contribute as an auxiliary support for language processing.

Actually, although some earlier studies on acute traumatic spinal cord injury (SCI) patients have documented the presence of cognitive deficits in different domains, such as attention, executive functioning, memory, and language (22–27), none of them reported sufficient evidence that the cognitive impairment was specifically related to SCI. Indeed, factors contributing to those deficits were varied. Some patients had concomitant traumatic brain injury at the time of their accidents (26). Others had a history of cerebral vascular insufficiency (27). In addition, the long-term cognitive effects of alcohol and substance abuse, which have been found to approach a prevalence of 50% in the SCI population, may have contributed to cognitive or behavioral disorders (24, 25).

Given the wide variability of cortical lesions among aphasic patients, it is not always easy to localize through non-invasive brain stimulation techniques, such as transcranial direct current stimulation (tDCS), the optimal stimulation cortical sites, unless we use additional very expensive methodologies, such as neuroimaging and/or modeling (28, 29). This points to the urgency of considering other vicarious systems, functionally connected to the brain, that, when stimulated, contribute to the recovery of language.

Indeed, it has been shown that the application of tDCS over the motor cortex influences brain excitability, and hence can also modulate the spinal cord (30–32). By developing a constant low-intensity current (1–2 mA) through two large electrodes located on the targeted areas, tDCS increases or reduces cortical excitability after anodal or cathodal stimulation, respectively, possibly by inducing depolarization or hyperpolarization of the neuronal membrane resting potential (33–36). Accordingly, in a series of experiments performed in healthy subjects, Roche et al. (37, 38) showed that anodal tDCS over the motor cortex modified spinal network excitability by increasing disynaptic inhibition of spinal motoneurons. The authors suggested that the increase of disynaptic inhibition relies on an increase of disynaptic interneuron excitability and that tDCS over the motor cortex in human subjects induces effects on the spinal network. Similarly, Di Lazzaro et al. (39) found that 20 min of anodal tDCS over the primary motor cortex lead to a pronounced increase of D wave. Since the D wave is produced by direct activation of corticospinal axons (39), the authors concluded that anodal tDCS induced changes in excitability in corticospinal projections. Indeed, in patients with SCI, spinal plasticity induced by anodal tDCS over the motor cortex may promote the effects of locomotor training through modulation of spinal interneurons (32). On the other hand, it has also been shown that spinal manipulation, in stroke patients who are recovering from muscle degrading dysfunctions, leads to changes in cortical excitability, as measured by significant larger movement related cortical potential amplitudes after the treatment (40).

Therefore, given this strong reciprocal connections between the cortex and the spinal cord, we might assume that the stimulation of the spinal cord could influence activity into the sensorimotor cortex, through the ascending spinal pathways, which, in turn, might facilitate language processing (4, 16–18, 41).

To our knowledge, the impact of transcutaneous spinal direct current stimulation (tsDCS) on language recovery has not been investigated so far. A number of studies have already used tsDCS for modulating spinal cord activity in humans along the lemniscal pathway and nociceptive spinal system [(42–44), see Ref. (45) for review]. In most of these studies, the active electrode was placed over the thoracic vertebrae (T10–T12) and the reference electrode above the right arm while the current (2–3 mA) was delivered for 20–30 min (42–44, 46). Different from tDCS studies in which the anode applied over the cortical areas increases cortical excitability (30, 39, 47), in those works, anodal tsDCS had an overall inhibitory effect on spinal cord activity, while cathodal tsDCS did not produce polarity-specific effects (42, 43, 48). Interestingly for our study, it has also been shown that anodal tsDCS might elicit neurophysiological changes into the brain, through the activation of tonic afferent systems to the cortex (46–49). Indeed, Bocci et al. (46) assessed changes in intracortical excitability in 10 healthy subjects following tsDCS (2 mA, 20 min) over the thoracic vertebrae (T9–T11 level) by evaluating changes in motor-evoked potentials (MEPs) recorded from the first digital interosseus and the tibialis anterior muscles. Results showed that tsDCS was able to modulate intracortical excitability in a polarity-specific manner. Anodal tsDCS decreased MEP amplitudes, while cathodal tsDCS elicited opposite effects. Similarly, the same authors found that anodal tsDCS (T9–T11 level, 2 mA, 20 min) increased transcallosal conduction time and interhemispheric delay in motor connectivity, leading to a functional disconnection between hemispheres (49).

Therefore, given that one of the main function of the spinal cord is to translate sensory information into motor output and that tsDCS exerts its influence also into the brain (46–49), it is possible that tsDCS contributes to the recovery of language, in particular for those words characterized by motor properties, such as action verbs (i.e., *to bite*). Conversely, nouns not typically related to specific action (i.e., *the cloud*) would not activate the motor pathways and, therefore, should not benefit of this facilitation (11, 12).

In the present study, 14 aphasic participants underwent a daily language treatment for their word retrieval difficulties while delivering tsDCS. To ensure that the effects were specific for spinal cord stimulation and, therefore, that we were acting as far as possible from the cortex, we chose to stimulate the thoracic level. Based on previous results (11, 12), we expected to find a beneficial effect on response accuracy and vocal reaction times only for verbs.

## Materials and Methods

### Participants

Fourteen chronic aphasic subjects (nine males and five females) who had suffered a single left hemisphere stroke were included in the study. Inclusion criteria were native Italian proficiency, pre-morbid right handedness, a single left hemispheric stroke at least 6 months prior to the investigation, and a mild comprehension impairment score equal to or greater than 14/36 (50). Subjects more than 75 years old with epileptic seizures, previous brain lesions, possible spinal cord comorbidities, and with any type of implanted electronic device (e.g., pacemaker) were excluded. None of the participants have received structured language therapy for at least 6 months before the time of inclusion in the study in order to prevent confounding therapy effects.

### Ethical Approval

The data analyzed in the current study were collected in accordance with the Helsinki Declaration and the Institutional Review Board of the IRCCS Fondazione Santa Lucia, Rome, Italy. Prior to participation, all patients signed informed consent forms.

### Clinical Data

**Figure 1:**
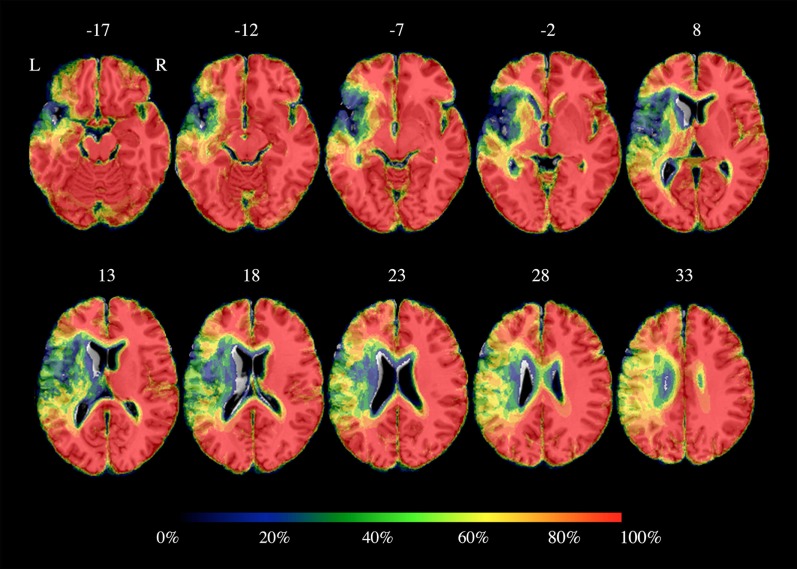
Percentage brain parenchyma overlap across patients. Color bar refers to the amount of spared voxels: 0% corresponds to total loss of cortical gray matter and 100% to preserved cortical gray matter. As shown, the highest damage was located into the left inferior frontal gyrus, the left temporal lobe and the left insula and it partially included the left precentral and postcentral gyri. Axial coordinates refer to the standard space (MNI152).

In all patients, the MRI revealed an ischemic lesion involving the left hemisphere (see Figure 1). The aphasic disorders were assessed using standardized language tests [the Battery for the Analysis of Aphasic Disorders, BADA test (51)]; Token Test (50). In order to ensure good cooperation during the treatment, subjects were recruited only if their score at the Token test revealed mild comprehension skills (score equal to or greater than 14/36). Subjects were also administered a memory test [i.e., digit span (52)] and a computerized Battery for Attentional Performance (53), which excluded the presence of working memory and attention deficits that might have confounded the data. The 14 subjects were classified as non-fluent aphasics because of their reduced spontaneous speech with short sentences and frequent word-finding difficulties. They had no articulatory deficits with preserved word repetition and reading. In a naming task, all patients had lexical retrieval difficulties [BADA test (51)] (see Table 1).

**Table 1:** Sociodemographic and clinical data of the 14 non-fluent aphasic patients.

P	G	Age (years)	Educ level (years)	Stroke type	Time post-onset	NN (%)	VN (%)	NC (%)	VC (%)	TT	Loss of gray matter volume within Broadmann areas (%)
1	F	71	10	ISCH	2 years, 1 month	30	32	100	100	14/36	BA 38 (90); BA 45 (76); BA 47 (52)
2	M	53	18	ISCH	6 years, 7 months	10	0	100	100	14/36	BA 21 (56); BA 38 (74); BA 44 (79); BA 47 (60)
3	M	57	13	ISCH	4 years, 4 months	25	20	100	100	15/36	BA 21 (69); BA 22 (91); BA 38 (70); BA 44 (52); BA 47 (61)
4	M	49	16	ISCH	8 years, 3 months	10	10	100	100	14/36	BA 1, 3 (50); BA 2 (67); BA 21 (60); BA 22 (64); BA 38 (88)
5	M	61	18	ISCH	1 year, 8 months	60	67	100	100	22/36	BA 1 (55); BA 47 (60)
6	M	46	8	ISCH	1 year, 6 months	15	20	100	100	14/36	BA 1 (70); BA 2 (66); BA 3 (62); BA 22 (57); BA 40 (59)
7	F	56	13	ISCH	8 years, 1 month	50	46	100	100	15/36	BA 1 (64); BA 2 (74); BA 3 (73); BA 22 (56); BA 38 (79); BA 39 (62); BA 40, 44, 45 (100); BA 46 (75); BA 47 (93)
8	M	68	18	ISCH	1 year, 6 months	60	64	100	100	22/36	BA 44 (76)
9	F	49	18	HEM	1 year, 7 months	57	57	100	100	14/36	BA 38 (100); BA 44 (90); BA 45 (68); BA 47 (84)
10	M	41	13	HEM	5 years, 4 months	53	54	100	100	14/36	BA 47 (60)
11	F	68	8	ISCH	8 years, 7 months	57	57	100	100	15/36	BA 20 (60); BA 21 (80); BA 22 (87); BA 38 (81)
12	M	51	8	HEM	1 year, 7 months	37	39	100	100	26/36	BA 1 (53); BA 2 (37)
13	F	74	12	ISCH	1 year, 8 months	30	32	100	100	15/36	BA 20 (60); BA 21 (80); BA 38 (81)
14	M	61	13	HEM	8 years	50	66	100	100	14/36	BA 20 (67); BA 21 (41); BA 38 (46); BA 44 (74); BA 47 (47)

*P, participants; G, gender; F, female, M, male; educ. Level, educational level; ISCH and HEM, ischemic and hemorrhagic stroke; Percentage of correct responses in NN, noun naming; VN, verb naming; NC, noun comprehension; VC, verb comprehension [BADA test (21)]; TT, token test, cutoff, 29/36 (22); BA 1-2-3, primary somatosensory cortex; BA 20, 21, 22, inferior, middle, and superior temporal gyri; BA 38, temporal pole; BA 39, 40, angular and supramarginal gyri; BA 44, 45, pars opercularis and triangularis (inferior frontal gyrus); BA 46, dorsolateral prefrontal cortex; BA 47, pars orbitalis (inferior frontal gyrus)*.

In summary, all patients were right handed before stroke, in a chronic phase, with a single left hemispheric stroke and no epileptic seizures. They were all classified as non-fluent aphasics with mild comprehension skills and no articulatory deficits.

### Materials

45 action verbs [15 related to hand (e.g., to knock), 15 to mouth (e.g., to bite), and 15 to body actions (e.g., to dance)] and 45 non-manipulable nouns (e.g., clouds) were used. Nouns and actions were matched for number of letters, surface frequency (54), imageability (estimated on the basis of a sample of 20 normal participants along a seven-point scale) and age of acquisition [estimated on the basis of a sample of 20 normal participants along a nine-point scale (55)]. Both imageability and age-of-acquisition ratings were collected by asking volunteers to judge printed words.

### Procedure

#### Transcutaneous Spinal Direct Current Stimulation

Transcutaneous spinal direct current stimulation was delivered using a battery driven Eldith (neuroConn GmbH Programmable Direct Current Stimulator, Germany) with a pair of surface-soaked sponge electrodes (5 cm × 7 cm). As in previous studies (46–49), a constant current of 2 mA intensity was applied through the active electrode on the 10th thoracic vertebra (spanned from the ninth to the 11th thoracic vertebrae) for 20 min while the reference electrode was placed over the right shoulder on the deltoid muscle (48). Indeed, several studies investigating corticospinal excitability (56–61) have suggested that longer-lasting robust effects are usually found with higher intensities (2 mA) (61) and longer (≥10 min) durations (61). Three different stimulation conditions were carried out: (1) anodic, (2) cathodic, and (3) sham. Sham stimulation was performed exactly like the other two conditions but the stimulator was turned off after 30 s (61). All patients underwent the three stimulation conditions whose order was randomized across subjects. To ensure the double-blind procedure, both the experimenter and the patient were blinded regarding the stimulation condition and the stimulator was turned on/off by another person. For each category (verbs vs. nouns), stimuli were subdivided into three lists of 15 items each matched for frequency, length, imaginability, and age of acquisition. For the three lists of verbs, each list included five hand-, five mouth-, and five body-related actions matched for the different variables. Both for the noun and the verb naming task, the assignment of each list to each stimulation condition (anodic vs. cathodic vs. sham) was randomized across conditions.

#### Word Retrieval Training

**Figure 2:**
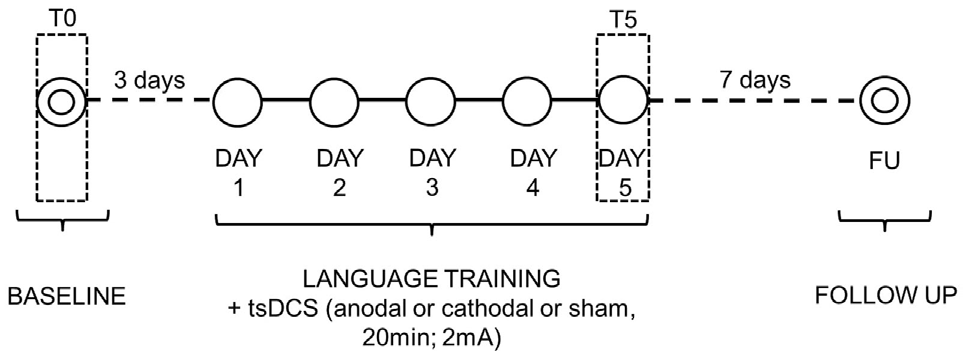
Overview of study design.

Once the electrodes were placed, subjects performed the naming task while they received 20 min of tsDCS. For each treatment, subjects were asked to name each picture that appeared on the PC screen (screen size 15″, viewing distance 1 m) for 20 s preceeded by a fixation point, which lasted 800 ms [see also Ref. (59) for similar procedure]. Only if the subject spontaneously correctly named the picture, the examiner manually recorded the response type on a separate sheet. If the subject failed or did not answer within 20 s, the corresponding written name was presented below the picture for 5 s and the subject was asked to read the word aloud. The pair of stimuli remained on the screen until the subject read the word or 5 s elapsed. In all cases, subjects were able to correctly read the word. Vocal reaction times were calculated from the presentation of the picture to the pronunciation of the first phoneme through *Audacity 2.1.2 Software*. Only the correctly pronounced words were considered. Each stimulation condition was performed in five consecutive daily sessions over three weeks with 6 days of intersession interval. The order of items presentation was randomized across sessions. To measure baseline performance, for each condition, three days before the training each subject was asked to name the pictures, one at a time, without help. At 1 week after each stimulation condition, all subjects were again shown the corresponding list of items and asked to name them without help. As before, the examiner manually recorded the answers (see Figure 2).

### Data Analysis

Data were analyzed with SPSS 17.0 software. Statistical analyses were performed with two separate analyses of variances for response accuracy and vocal reaction times with three within-subject factors [WORDS CATEGORY (nouns vs. verbs)], TIME [baseline (T0) vs. end of training (T5) vs. follow-up (FU)] and CONDITION (anodal vs. cathodal vs. sham). If the analysis of variance (ANOVA) showed significant effects, respective *post hoc* Bonferroni tests were conducted. For all analyses, *p*-values < 0.05 were considered as statistically significant. All subjects well tolerated the experiment and none reported side effects or adverse reaction to the protocol.

## Results

### Accuracy

The analysis showed a significant effect of WORD CATEGORY [*F*(1, 13) = 36.60, *p* < 0.001], TIME [*F*(2, 26) = 102.92, *p* < 0.001], and CONDITION [*F*(2, 26) = 6.61, *p* = 0.005]. Overall, the percentage of correct responses for nouns was greater compared to verbs (nouns: mean = 59%, SD = 27 vs. verbs: mean = 47%, SD = 25, *p* < 0.001) and the percentage of correct responses was greater at the end of training (T5) compared to the baseline (T0) (T5: mean = 66%, SD = 24 vs. T0: mean = 32%, SD = 19, *p* < 0.001) and in the anodal tsDCS condition compared to the other two conditions (anodal: mean = 57%, SD = 28 vs. cathodal: mean = 51%, SD = 26 vs. sham: mean = 51%, SD = 26, *p* = 0.005).

The interaction WORD CATEGORY × CONDITION × TIME was also significant [*F*(4, 52) = 4.66, *p* = 0.003]. Indeed, while no significant differences between nouns and verbs were found for all experimental conditions at T0 (differences between nouns vs. verbs, anodal = 6%, *p* = 1; cathodal = 2%, *p* = 1; sham = 6%, *p* = 1), verbs significantly improved in all conditions at the end of treatment (T5) with respect to the baseline (T0) (difference between T5 and T0 anodal = 42%, *p* < 0.001; cathodal = 20%, *p* < 0.001; sham = 22%, *p* < 0.001) but the improvement was greater only after the anodal tsDCS condition compared to the other two conditions, which did not differ from each other (anodal vs. cathodal = 18%, *p* < 0.001; anodal vs. sham = 18%, *p* < 0.001; cathodal vs. sham = 0%, *p* = 1). No specific trend was found due to the properties of the verb itself. Indeed, at the end of treatment, the mean number of correct responses produced in the anodal condition did not reveal any significant differences among the different verb categories (hand vs. mouth vs. body actions) [*F*(2, 26) = 0.02, *p* = 0.98].

On the contrary, although the percentage of correct responses for nouns significantly improved in all conditions at the end of treatment (T5) with respect to baseline (T0) (difference between T5 and T0 anodal = 44%, *p* < 0.001; cathodal = 40%, *p* < 0.001; sham = 39%, *p* < 0.001), no significant differences between the three conditions were found (anodal vs. cathodal = 4%, *p* = 1; anodal vs. sham = 3%, *p* = 1; cathodal vs. sham = −1%, *p* = 1).

**Figure 3:**
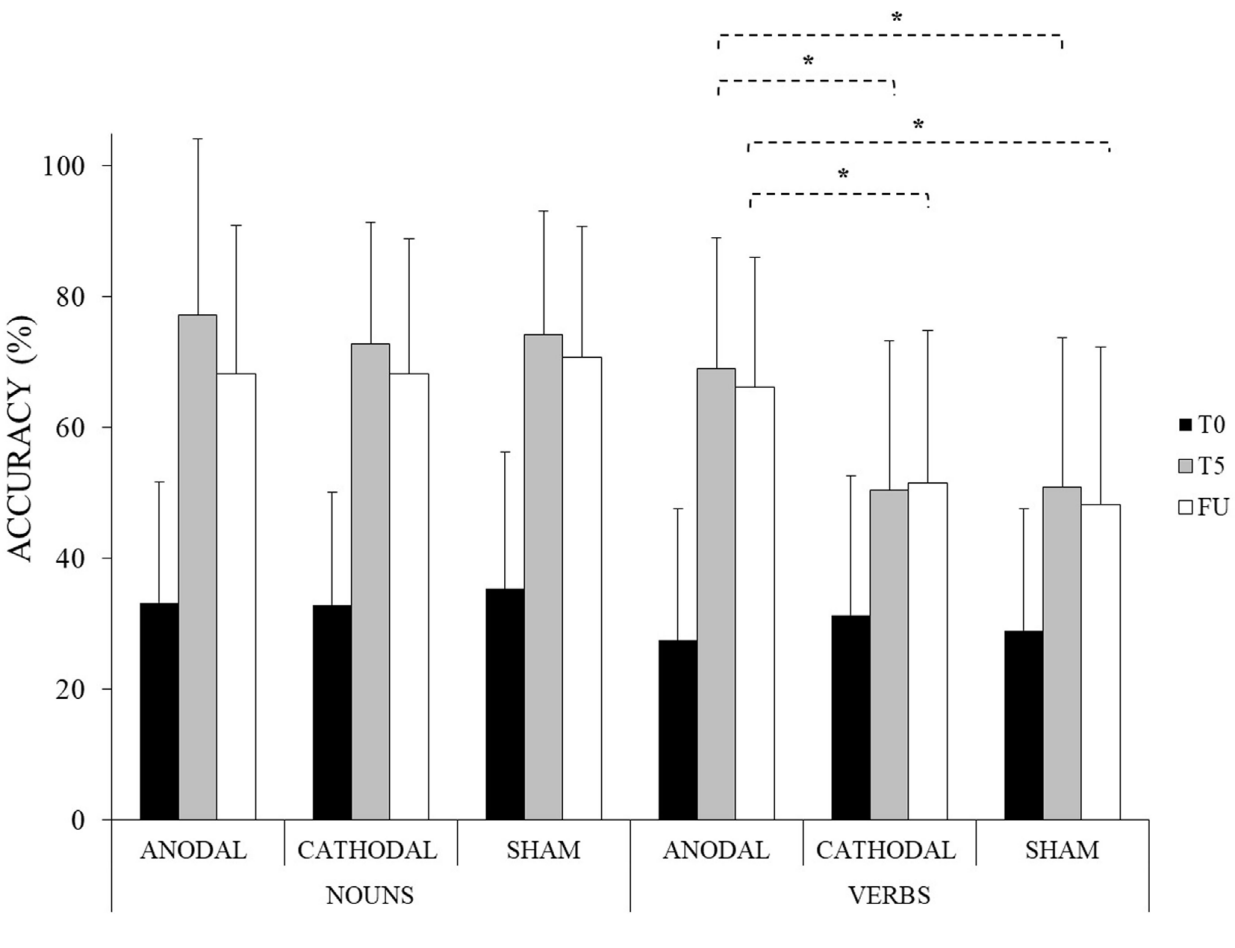
Mean percentage of response accuracy for nouns and verbs at baseline (T0), at the end of the treatment (T5) and at follow-up (FU) for the anodal, cathodal and sham condition, respectively (*<0.001). Error bars represent SD.

Moreover, the improvement reached for verbs in the anodal condition persisted at 1 week after the treatment (anodal FU-T5 = −3%, *p* = 1) and it remained greater compared to the other two conditions (anodal vs. cathodal at FU = 14%, *p* < 0.001; anodal vs. sham at FU = 18%, *p* < 0.001; cathodal vs. sham at FU = 4%, *p* = 1) (see Table 2; Figure 3).

**Table 2:** Mean percentage of correct responses in noun and verb naming for anodal, cathodal, and sham condition for each patient.

P	Order of cond.	Nouns accuracy (%)									Verbs accuracy (%)								
		Anodal			Cathodal			Sham			Anodal			Cathodal			Sham		
		T0	T5	Follow-up (FU)	T0	T5	FU	T0	T5	FU	T0	T5	FU	T0	T5	FU	T0	T5	FU
1	a-c-s	35	45	40	20	40**	50	25	40***	35	23	40**	35	23	20	19	26	23	19
2	c-a-s	15	50*	50	20	65*	50	30	70*	50	0	55*	50	3	39*	39	0	39*	35
3	a-c-s	30	85*	50	30	70*	60	45	70*	70	16	42*	45	17	39*	35	23	35	26
4	c-a-s	5	25*	30	15	60*	30	10	50*	55	0	36*	30	6	19**	39	0	19*	19
5	s-a-c	60	80**	60	70	85***	80	70	90*	90	35	84*	78	64	71	71	48	77*	65
6	s-c-a	5	35*	55	10	40*	35	0	50*	45	3	68*	65	13	16	19	6	26*	32
7	c-a-s	55	95*	95	35	80*	85	50	95*	90	35	80*	84	42	65**	74	45	71*	74
8	c-a-s	65	100*	100	60	100*	90	60	100*	100	71	100*	100	65	94*	94	48	87*	100
9	c-a-s	40	75*	60	50	60	70	60	85*	75	42	81*	71	45	61***	61	45	65**	55
10	s-a-c	30	100*	85	35	80*	80	40	85*	65	39	84*	80	42	58***	42	29	40	45
11	s-c-a	35	100*	100	40	100*	100	40	95*	100	39	90*	80	19	65*	81	39	71*	68
12	a-s-c	20	90*	70	20	85*	75	10	70*	70	10	58*	61	10	40*	35	10	30*	30
13	a-c-s	30	100*	70	25	75	70	20	60*	65	30	74*	70	35	50	45	35	65*	68
14	s-a-c	40	100*	90	30	80*	80	35	80*	80	42	81*	77	55	70***	68	52	65	39
Mean		33	77	68	33	73	68	35	74	71	27	69	66	31	51	52	29	51	48
SD		18	27	23	17	19	21	21	19	20	20	20	20	21	23	23	19	23	24

P, participants; order of cond, order of conditions, bold font is necessary to highlight the significant differences in the Chi Square Test, * < 0.001; ** ≤ 0.01; *** < 0.05.

We further explored whether the group statistical patterns hold true for all individuals in the study. Thus, for each subject, we measured the amount of improvement at the end of treatment in all conditions (anodal vs. cathodal vs. sham) for the verb and noun category, respectively. For the verb category, the pattern resulted consistent in 11 subjects out of 14 showing a greater improvement in the anodal condition compared to the other two (P1-2-4-5-6-7-9-10-12-13-14). The two remaining subjects showed a greater improvement in the anodal condition compared to sham but not compared to cathodal condition (P3 and 11). Only one subject (P8) showed no significant differences between all conditions. For the noun category, the pattern resulted consistent in 8 subjects out of 14 showing no significant differences between the three conditions (P1-2-5-7-8-11-12-14). Four out of 14 subjects (P3-9-10-13) showed a greater improvement for the anodal condition compared to the sham and/or cathodal condition while, the two remaining subjects showed a greater improvement for the sham and/or cathodal condition compared to the anodal one (P4 and 6) (Chi square tests: *p* < 0.05 for all significant comparisons). Thus, the case series analysis mostly confirmed the group analysis results showing a behavioral pattern more homogenous and consistent across subjects for the verb than for the noun category.

In order to investigate if tDCS had a different impact on the subject’s response, we classified the errors made by each subject in all experimental conditions. As shown in Table 3, errors were (1) no responses, (2) semantic paraphasias, and (3) unrelated verb responses but, at baseline (T0), for both the noun and the verb naming, errors were predominantly “no responses.” Thus, we conducted an ANOVA on the number of “no responses” with three within-subject factors: TASK (noun naming vs. verb naming), CONDITION (anodal vs. cathodal vs. sham), and TIME [baseline (T0) vs. end of the treatment (T5)]. The analysis revealed a significant interaction TASK × CONDITION × TIME [*F*(2, 26) = 6.66, *p* = 0.005]. Indeed, although all experimental conditions led to a lower number of “no responses” at the end of treatment (T5) compared to the baseline (T0) (noun naming: difference between T5 and T0 anodal = 5, *p* < 0.001; cathodal = 5, *p* < 0.001; sham = 6, *p* < 0.001; verb naming: difference between T5 and T0 anodal = 6, *p* < 0.001; cathodal = 4, *p* < 0.001; sham = 4, *p* < 0.001), at the end of treatment (T5), only in the verb naming task the number of “no responses” was lower after anodal stimulation compared to the other two conditions which did not differ from each other (anodal vs. cathodal = −2, *p* < 0.001; anodal vs. sham = −2, *p* < 0.001; cathodal vs. sham = 0, *p* = 1). Thus, these results resembled those previously found for the accuracy data.

**Table 3:** Mean number of errors in noun and verb naming task at baseline (T0) and at the end of treatment (T5) for each transcutaneous spinal direct current stimulation conditions (±SD).

	Noun naming						Verb naming					
	No responses		Semantic paraphasias		Unrelated responses		No responses		Semantic paraphasias		Unrelated responses	
	T0	T5	T0	T5	T0	T5	T0	T5	T0	T5	T0	T5
Anodal	8 (±3)	3 (±3)	1 (±1)	0 (±1)	1 (±1)	0 (±1)	10 (±4)	4 (±3)	1 (±1)	1 (±1)	1 (±1)	1 (±1)
Cathodal	8 (±3)	3 (±3)	1 (±1)	1 (±1)	1 (±1)	1 (±1)	10 (±4)	6 (±3)	1 (±1)	1 (±1)	0 (±0)	0 (±1)
Sham	8 (±4)	2 (±3)	1 (±1)	1 (±1)	1 (±1)	1 (±1)	10 (±3)	6 (±3)	1 (±1)	1 (±1)	1 (±1)	1 (±1)

### Vocal Reaction Times

The analysis showed a significant effect of WORD CATEGORY [*F*(1, 13) = 33.05, *p* < 0.001] and TIME [*F*(2, 26) = 48.74, *p* < 0.001]. Overall, participants were significantly faster to name nouns compared to verbs (nouns: mean = 10,656 ms, SD = 5,327 vs. verbs: mean = 13,106 ms, SD = 4441, *p* < 0.001) and vocal reaction times were faster at the end of training (T5) compared to baseline (T0) (T5: mean = 10,139 ms, SD = 5,204 vs. T0: mean = 15,132 ms, SD = 2905, *p* < 0.001).

The interaction WORD CATEGORY × CONDITION × TIME was also significant [*F*(4, 52) = 3.49, *p* = 0.01]. Indeed, while no significant differences were found between the two categories for all experimental conditions at T0 (differences between nouns vs. verbs, anodal = −1,104 ms; *p* = 1; cathodal = −837 ms, *p* = 1; sham = −1,092 ms, *p* = 1), vocal reaction times were significantly faster for verbs in all conditions at the end of treatment (T5) with respect to the baseline (T0) (difference between T5 and T0 anodal = −5,195 ms, *p* < 0.001; cathodal = −2,856 ms, *p* < 0.001; sham = −3,258 ms, *p* < 0.001) but the improvement was greater only after the anodal tsDCS condition compared to the other two conditions, which did not differ from each other (anodal vs. cathodal = −2,269 ms, *p* < 0.001; anodal vs. sham = −2,026 ms, *p* = 0.001; cathodal vs. sham = 243 ms, *p* = 1). On the contrary, although vocal reaction times were significantly faster for nouns in all conditions at the end of treatment (T5) with respect to the baseline (T0) (difference between T5 and T0 anodal = −6,218 ms, *p* < 0.001; cathodal = −5,574 ms, *p* < 0.001; sham = −6,854 ms, *p* < 0.001), no significant differences between the three conditions were found (anodal vs. cathodal = −841 ms, *p* = 1; anodal vs. sham = 535 ms, *p* = 1; cathodal vs. sham = 1,376 ms, *p* = 0.23).

**Figure 4:**
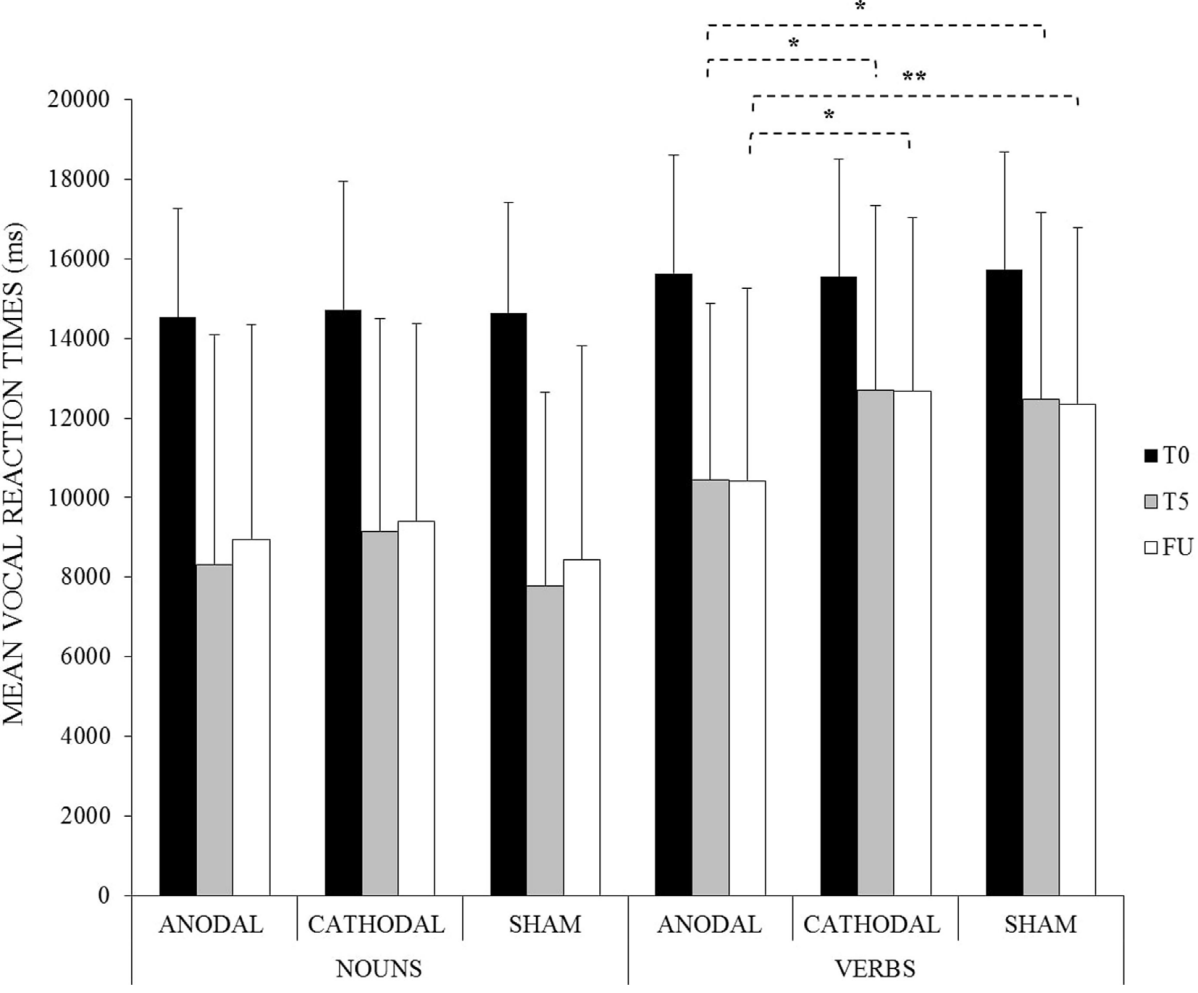
Mean vocal reaction times for noun and verb naming at baseline (T0), at the end of the treatment (T5) and at follow-up (FU) for the anodal, cathodal, and sham condition, respectively (*<0.001, **<0.01). Error bars represent SD.

Moreover, the improvement reached for verbs in the anodal condition persisted at 1 week after the treatment (anodal FU-T5 = −28 ms, *p* = 1). Indeed, vocal reaction times were still significantly faster compared to the other two conditions (anodal vs. cathodal at FU = −2,276 ms, *p* < 0.001; anodal vs. sham at FU = −1,936 ms, *p* = 0.003; cathodal vs. sham at FU = 304 ms, *p* = 1) (see Figure 4).

In order to control for possible influence of age, educational level, and time post-onset variables on the amount of improvement found for verb naming, correlational analyses were also conducted. Both for accuracy and reaction time data, no significant correlations were observed (*p* > 0.05). No differences between males and females emerged as well (unpaired *t*-test, *p* > 0.05).

### Further Analysis

It may be argued that the observed results were an artifact of treatment efficacy, which was greater for nouns than for verbs. In this case, in the sham condition, subjects would have enough time to reach the same amount of improvement found in the anodal condition, resulting in no significant differences between the two conditions for the noun category. Thus, we reasoned that if this would be the case, we should have found different treatment effects across sessions (T0, T1, T2, T3, T4, T5) in the sham condition for nouns and verbs, respectively. However, statistical analyses revealed no significant differences (mean percentage of improvement for nouns: T1–T0 = 9%, T2–T1 = 9%, T3–T2 = 8%, T4–T3 = 6%, T5–T4 = 6% mean percentage of improvement for verbs: T1–T0 = 7%, T2–T1 = 6%, T3–T2 = 5%, T4–T3 = 1%, T5–T4 = 4%, *t*-tests, *p* > 0.05).

## Discussion

This study assessed whether tsDCS coupled with language treatment improves word retrieval in persons with chronic non-fluent aphasia. Our findings showed that anodal tsDCS differently affected the amount of improvement in noun and verb naming. Indeed, while noun and verb naming significantly improved in all patients for each condition at the end of training due to language treatment, anodal tsDCS boosted recovery only for verbs. Moreover, FU testing showed that these effects lasted over 1 week after the intervention. This specificity argues against an effect simply due to enhanced cognitive arousal which should have influenced both verb and noun naming.

Although this finding seems surprising, it suggests that the spinal cord takes part in verb processing acting as a “bridge” for conveying tsDCS induced changes into brain networks. Thereby inducing neuromodulation effects into brain areas involved in verb naming.

As previously stated, opposite excitability changes induced by cortical tDCS (60, 61) and spinal tDCS (42, 44) of the same polarity have been reported. Indeed, while anodal tDCS is generally facilitatory to the cortex (30, 39, 47), anodal tsDCS was found to decrease the amplitude of the somatosensory evoked potentials (42), laser evoked potentials (44) and motor responses evoked by transcranial magnetic stimulation (46) due to an hyperpolarization of the axons running along the spinal columns, while cathodal stimulation did not exert any influence (42, 46). It has also been shown that thoracic tsDCS elicits intracortical changes through the thalamus and the reticular systems (46, 49). Indeed, recent modeling studies have proved that, despite some inter-individual differences, the electric field induced by thoracic tsDCS is longitudinally directed along the vertebral column, especially when the return electrode is placed over the right arm (62, 63). Yet, the electric field induced by thoracic tsDCS is maximum at thoracic level and it can increase the somatosensory activity from the spinal cord to the brain (62, 63).

Even if the exact underlying tsDCS mechanisms over the corticospinal system, in our study, remain largely speculative, the hypothesis might be advanced that anodal tsDCS has inhibited the tonic ascending system to the cortex, ultimately decreasing the activity into the sensorimotor areas. Paradoxically, the resulting reduction could have potentiated their function. Indeed, the hypothesis has been advanced that inhibitory current might decrease the excitability of cortical inhibitory interneurons (64, 65), thus improving the efficacy of their related areas. Following previous suggestion, we might also hypothesize that anodal tsDCS has increased the interhemispheric delay in motor connectivity (49), thus, enhancing the functionality of the left sensorimotor cortices through inhibition of its right homologs (66). Indeed, the model of interhemispheric competition between the residual language areas in the left-damaged hemisphere and the intact right hemisphere (akin to models of motor recovery after stroke) proposes that in patients with left hemispheric damage, the homotopic contralateral right hemispheric areas may be in a state of abnormally high activation and may exert an inhibitory effect over the damaged hemisphere (67, 68). Thus, improvement may be possible either by increasing the output of the perilesional left hemisphere through excitatory stimulation (59, 69–71) or decreasing the inhibition from the intact right hemisphere by applying inhibitory current over the contralesional cortex (72–75). Therefore, in our study, we might hypothesize that tsDCS has inhibited the right hemisphere areas increasing the left hemisphere activity and, in particular, the left sensorimotor areas, making them more efficient. Indeed, all patients had preserved motor cortex and the amount of improvement obtained for verbs in the anodal condition was similar across patients with a partial damage over the somatosensory areas (see Table 1) and the rest of the group [five damaged subjects: mean = 49% (SD = 12) *p* < 0.001 vs. nine undamaged subjects: mean = 38% (SD = 11) *p* < 0.001; difference between the two groups: mean = 11%, unpaired *t*-test *p* = 0.14].

An effect of tsDCS on pharmacologically defined systems cannot also be ruled out. Indeed, it has been shown that the mechanisms of action underlying tDCS could also involve receptors and neurotransmitters. For instance, neurotransmitters such as GABA and glutamate undergo substantial changes into the brain after cortical tDCS over the motor cortex (47, 76). Accordingly, Stagg et al. (76) found that excitatory (anodal) tDCS reduced GABA levels within the sensorimotor cortices while inhibitory (cathodal) stimulation reduced glutamatergic neuronal activity with a highly correlated reduction in GABA over the same region, due to the close biochemical relationship between the two neurotransmitters. A local decrease of GABA levels in the sensorimotor cortex is strongly related with synaptic plasticity and it is positively correlated with an improvement in motor learning (77–79). Although this effect might be task specific, one further hypothesis to consider is that, in our study, the inhibitory current delivered through anodal tsDCS has decreased both glutamate and GABA levels into the sensorimotor cortices leading to an improvement of their function (76, 78, 79).

All of the above mentioned hypotheses explain the specificity of anodal tDCS for verbs. As stated in the Introduction, several lines of evidence have already shown that action words are partly represented into the sensorimotor cortex due to their sensorimotor semantic properties which, in turn, are involved in action understanding and naming (4, 10–13, 15–18, 41). This implies that the sensorimotor cortex may also process action concepts (6). Yet, Repetto et al. (80) found that processing hand-related action words, but not abstract words, was impaired after repetitive TMS to the left primary motor cortex [see also Ref. (81)]. Similarly, very recently, Meinzer et al. (82) have shown that anodal tDCS delivered over the left motor cortex with concomitant language training improved naming abilities in a group of 26 patients with chronic aphasia.

Thus, we hypothesize that anodal tDCS has influenced neural activity along the ascending spinal pathways, ultimately modulating activity in the sensory–motor cortex. This has, in turn, facilitated verb retrieval. Indeed, the improvement found in verb naming was not specific for any type of action. Yet, the current delivered over the thoracic vertebrae going up to the brain also resulted in an improvement on verbs (i.e., hand and mouth actions) whose corresponding muscles are innervated above the stimulated region. This last result further confirms our hypothesis that action verbs were ultimately processed by the sensorimotor areas. On the contrary, since nouns do not express a motor content they did not benefit from stimulation.

In conclusion, although other studies will further elucidate our understanding on the role of the spinal cord in language processing, we believe that our results are promising since, for the first time, they suggest that spinal tDCS significantly affects the sensorimotor system removing the need to establish which part of this system should be targeted with tDCS. Since verbs play a crucial role in sentence construction which is essential to enhance speech production in persons with aphasia, we believe that this finding is important for treatment outcomes.

## Ethics Statement

This study was carried out in accordance with the recommendations of the Helsinki Declaration and the Institutional Review Board of the IRCCS Fondazione Santa Lucia, Rome, Italy with written informed consent from all subjects. All subjects gave written informed consent in accordance with the Declaration of Helsinki. The protocol was approved by the Institutional Review Board of the IRCCS Fondazione Santa Lucia, Rome, Italy.

## Author Contributions

Conceived and designed the experiment: PM, VF, and AP. Performed the experiment: VF and JS. Analyzed the data: VF, TG, and GC. Wrote the paper: PM. Edited the manuscript: PM, CC, and AP.

## Conflict of Interest Statement

The authors declare that the research was conducted in the absence of any commercial or financial relationships that could be construed as a potential conflict of interest.
